# Determination of Safe Corridors for External Fixation Pin Insertion in the Distal Long Bones: An Ex Vivo Anatomical Study in Neonatal Simmental Calves

**DOI:** 10.3390/vetsci13050475

**Published:** 2026-05-14

**Authors:** Kamil Serdar İnal, Can Nacar

**Affiliations:** 1 Department of Surgery, Faculty of Veterinary Medicine, University of Ondokuz Mayıs, 55200 Samsun, Turkey; 2 Department of Wild Animal Diseases, Faculty of Veterinary Medicine, University of Ondokuz Mayıs, 55200 Samsun, Turkey; can.nacar@omu.edu.tr

**Keywords:** biologic osteosynthesis, calf, external skeletal fixation, fracture, safe corridors

## Abstract

External skeletal fixation (ESF) is a versatile method for fracture management in neonatal calves but confers a significant risk of iatrogenic injury to vital structures. The aim of this ex vivo study was to systematically define safe, hazardous, and unsafe corridors for transcortical pin placement in the distal long bones (radius-ulna, tibia, metacarpus, and metatarsus) of Simmental calves to provide precise anatomical guidance. Six fresh Simmental calf cadavers without orthopaedic problems were included in the study. The forearm and hindlimb were disarticulated from the shoulder and hip joints, respectively. The radius-ulna, tibia, and metacarpal and metatarsal bones were dissected transversely from five anatomical levels. Safe, hazardous, and unsafe corridors were determined in each section. No safe corridor was found in the proximal metaphysis and proximal diaphysis of the radius-ulna. Safe corridors were found on the medial side of the radius-ulna, from the mid-diaphysis to the distal metaphysis. The metacarpal and metatarsal bones showed lateral and medial safe corridors. The tibia provided a continuous medial safe corridor. In conclusion, precise topographic mapping is vital for safe ESF. While anatomically safe corridors are the primary choice for ESF, hazardous corridors are viable alternatives if meticulously utilized. By contrast, unsafe corridors must be strictly avoided to prevent severe iatrogenic trauma.

## Simple Summary

Fracture management in newborn calves often involves using external fixation frames with pins inserted into the bone. While effective, placing these pins can carry a risk of accidentally damaging important blood vessels and nerves. This study sought to outline potential safe, hazardous, and unsafe corridors for inserting pins into the lower leg bones of Simmental calves. By examining the cross-sectional anatomy of deceased calves, we identified specific pathways for pin placement. Our observations suggest that the upper part of the forelimb bone may lack completely safe areas, whereas other sections appear to have specific safe zones on their inner or outer sides. In conclusion, having an anatomical reference can be highly beneficial for fracture repair. These findings serve as a helpful guide for veterinarians to potentially minimize further injury during surgery. Ultimately, this research provides value to society by improving surgical success and minimizing complications, ensuring a better quality of life and improved welfare for injured calves.

## 1. Introduction

Limb fractures are common in newborn calves owing to the fragility of the neonatal skeletal system. The most common aetiologies of limb fracture include interventions during dystocia and trauma resulting from the cow stepping on the calf’s limb [1,2].

Many stabilization methods for managing fractures in calves have been described, ranging from external coaptation to internal fixation [3,4,5]. Owing to its cost-effectiveness, cast immobilization is commonly used as a fracture treatment in ruminants. However, it has significant limitations and is often an unsuitable stabilization method for upper long bone fractures, such as those in the proximal or mid-diaphyseal regions of the radius or tibia [5,6]. Open reduction and internal fixation are also rarely performed because of practical constraints [6]. Consequently, external skeletal fixation (ESF) and the transfixation pin casting technique have emerged as excellent and versatile alternatives [6,7,8,9]. These techniques, including type Ia, type Ib, and circular configurations, are well documented and particularly effective for managing complex diaphyseal and metaphyseal fractures across the limbs [5,10,11,12].

Although ESF is a clinically well-established technique in calves [5,13], its success depends heavily on avoiding iatrogenic injuries. The most frequently reported complications in ruminants are pin tract infection and premature pin loosening due to thermal necrosis [5]. However, a critical but less discussed complication relates to the anatomical trajectory of transcortical pin placement [14]. Penetrating major neurovascular structures or passing pins through large muscle groups during insertion can lead to severe postoperative pain, reduced limb usage, and catastrophic functional losses [6]. Therefore, precise knowledge of cross-sectional anatomy is required to navigate transcortical pins safely and avoid these vital structures [15,16,17].

To mitigate these risks, surgeons rely on safe corridors and anatomical windows that allow pin placement without damaging any musculotendinous and neurovascular structures. In small animal orthopaedics (dogs and cats), the mapping of these corridors has successfully guided clinical practice by standardizing safe pin trajectories and optimizing ESF frame selection [16,17,18]. In contrast, a systematic, level-by-level anatomical map specifically tailored for neonatal calves is currently lacking. Although traditional bovine anatomy texts offer general topographic descriptions of adult cattle, they do not provide this targeted cross-sectional guidance.

Given the high clinical incidence of long bone fractures in this specific age group, this knowledge gap is critical [1]. Consequently, despite ESF being a standard procedure, pin placement in calves often relies on estimation rather than precise anatomical guidance. Therefore, to directly address this literature gap, the primary purpose of this ex vivo study was to provide the first systematic topographic reference by defining safe, hazardous, and unsafe corridors for pin placement in the distal long bones of the forelimbs and hindlimbs of neonatal calves.

## 2. Materials and Methods

### 2.1. Study Design

This ex vivo anatomical study was designed to define safe corridors for ESF and transfixion pin casting in bovine limbs. The study workflow followed a sequential process: collection of cadaveric specimens, radiographic evaluation to exclude pathologies, preparation of anatomical cross-sections, and morphometric analysis of neurovascular structures relative to the bone to determine safe insertion zones in the radius-ulna, tibia, and metacarpal and metatarsal bones.

### 2.2. Animals and Specimen Collection

Six male neonatal Simmental calves (6 forelimbs and 6 hindlimbs), with a mean age of 43.2 ± 9.5 days and a mean weight of 56.7 ± 7.5 kg, were included in the study. Their cadavers were obtained from the clinics of the Faculty of Veterinary Medicine. The animals originated from multiple breeding facilities in the region. The animals died of causes unrelated to the musculoskeletal system (e.g., respiratory or enteric diseases). The inclusion of exclusively male Simmental calves was coincidental, reflecting the clinical admissions during the study period rather than deliberate sex-based selection. The strict inclusion criteria for this study were as follows: aged 1–2 months, died from a cause unrelated to this study, had no musculoskeletal pathologies, and a post-mortem interval of less than 3 h. Immediately after death, the intact carcasses were stored in a +4 °C refrigerator. To strictly preserve tissue integrity, limb specimens were collected within 3 h post-mortem. Informed consent was obtained from the owners prior to the enrolment of the cadavers in the study. Orthogonal radiographic views (mediolateral and craniocaudal/dorsoplantar) were obtained specifically for each bone segment using a digital veterinary X-ray system (838 UHF100) equipped with a flat panel detector (Fujifilm FDR D-EVO SE Lite C35, Tokyo, Japan) and FVS-100 software (Fujifilm, Tokyo, Japan) to exclude any skeletal pathology.

### 2.3. Preparation of Anatomical Cross-Sections

All forelimbs and hindlimbs were disarticulated at the shoulder and hip joints, respectively. The right limbs were designated for transverse sectioning and cross-sectional anatomical evaluation, while the left limbs were used for topographic dissection to confirm neurovascular and musculotendinous structures [16,17,18]. After the skin of all limbs was removed, the right limbs were frozen at −20 °C for 24 h, whereas the left limbs were kept at 4 °C. To avoid disturbing or displacing the anatomical structures during the freezing process, a rope was secured with a slip knot proximal to the coronary band and tied to an S-shaped hook to allow the limbs to be frozen in a hanging, fully extended position.

**Figure 1:**
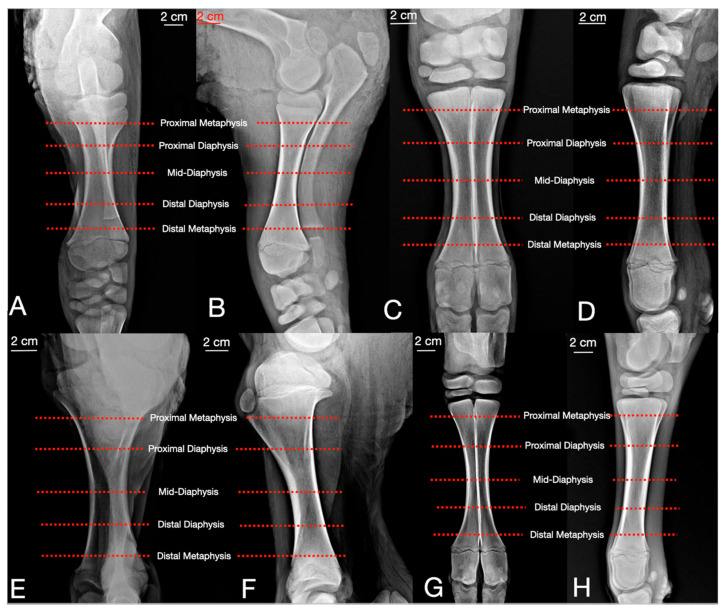
View of the transverse cross-sectional levels of the radius-ulna (**A**,**B**), metacarpal bones (**C**,**D**), tibia (**E**,**F**) and metatarsal bones (**G**,**H**) on mediolateral and craniocaudal radiographs in calves.

Each frozen right limb specimen was transversely sectioned into five levels using a band saw (Sönmez Endüstriyel, Istanbul, Turkey) [16,17]. The locations of these five transverse bone cuts were strategically standardized to evaluate clinically relevant pin placement sites and the comprehensive cross-sectional anatomy of the long bones (Figure 1). These levels were defined proportionally relative to the total bone length (0% = proximal articular surface, 100% = distal articular surface). The first and fifth sections were established at the proximal and distal metaphyseal limits (corresponding to approximately 15% and 85% of the total bone length, respectively). These specific locations represent the most anatomically feasible and clinically recommended sites for transfixation pin placement. The three intermediate sections were distributed at equal intervals between these metaphyseal limits (approximately 32.5%, 50%, and 67.5% of the bone length) to provide a continuous anatomical evaluation throughout the diaphyseal region (proximal, mid, and distal diaphysis). Each cross-sectional surface was photographed using a digital camera (FinePix S2500HD, Fujifilm Corporation, Tokyo, Japan).

### 2.4. Classification of Anatomical Corridors

Marti and Miller’s (1994) definitions of safe, hazardous, and unsafe corridors were adopted [16,17]. In this ex vivo model, important neurovascular structures were explicitly dichotomized based on established veterinary anatomical nomenclature and standard topographic texts [19,20]. Structures were classified as ‘major’ if they represented the primary axial vessels responsible for the main arterial supply and deep venous drainage of the distal limb (e.g., median artery, cranial tibial artery), or if they were primary nerve trunks providing essential motor innervation to large muscle groups (e.g., radial and tibial nerves). Conversely, structures were classified as ‘minor’ if they were terminal cutaneous sensory branches or small superficial vessels that possessed abundant collateral circulation.

**Figure 2:**
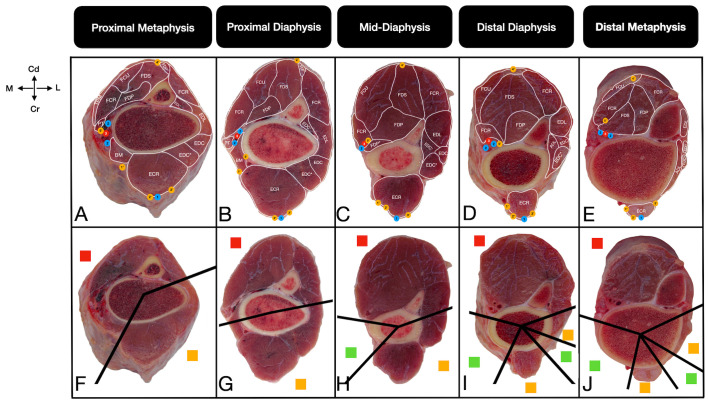
Transverse cross-sectional views of the anatomical (**A**–**E**) and pin placement corridors (**F**–**J**) in the radius-ulna of the calf at the proximal metaphysis (**A**,**F**), proximal diaphysis (**B**,**G**), mid-diaphysis (**C**,**H**), distal diaphysis (**D**,**I**), and distal metaphysis (**E**,**J**). The straight lines (**F**–**J**) show the boundaries of the pin placement corridors. The red squares indicate unsafe corridors, the yellow squares indicate hazardous corridors, and the green squares indicate safe corridors. Cr: Cranial, Cd: Caudal, M: Medial, L: Lateral. For a complete list of anatomical abbreviations, please refer to Supplementary Table S1.

**Figure 3:**
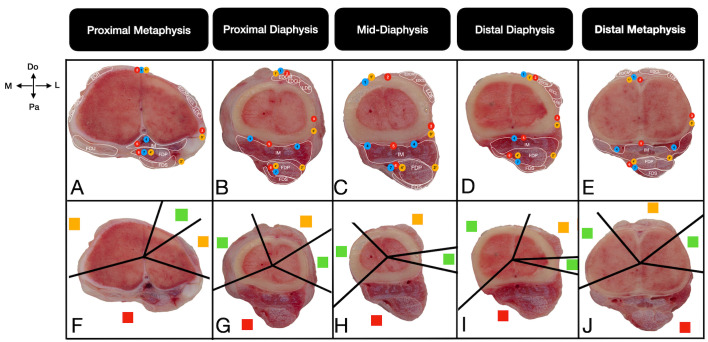
Transverse cross-sectional views of the anatomical (**A**–**E**) and pin placement corridors (**F**–**J**) in the metacarpal bones of the calf at the proximal metaphysis (**A**,**F**), proximal diaphysis (**B**,**G**), mid-diaphysis (**C**,**H**), distal diaphysis (**D**,**I**), and distal metaphysis (**E**,**J**). The straight lines (**F**–**J**) show the boundaries of the pin placement corridors. The red squares indicate unsafe corridors, the yellow squares indicate hazardous corridors, and the green squares indicate safe corridors. Do: Dorsal, Pa: Palmar, M: Medial, L: Lateral. For a complete list of anatomical abbreviations, please refer to Supplementary Table S1.

**Figure 4:**
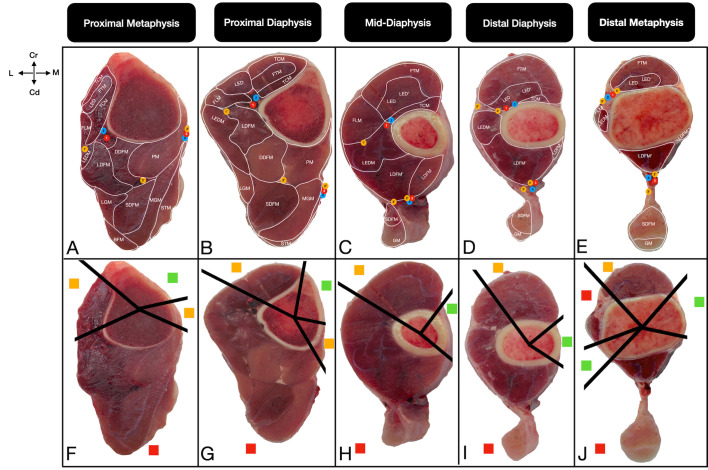
Transverse cross-sectional views of the anatomical (**A**–**E**) and pin placement corridors (**F**–**J**) in the tibia of the calf at the proximal metaphysis (**A**,**F**), proximal diaphysis (**B**,**G**), mid-diaphysis (**C**,**H**), distal diaphysis (**D**,**I**), and distal metaphysis (**E**,**J**). The straight lines (**F**–**J**) show the boundaries of the pin placement corridors. The red squares indicate unsafe corridors, the yellow squares indicate hazardous corridors, and the green squares indicate safe corridors. Cr: Cranial, Cd: Caudal, M: Medial, L: Lateral. For a complete list of anatomical abbreviations, please refer to Supplementary Table S1.

**Figure 5:**
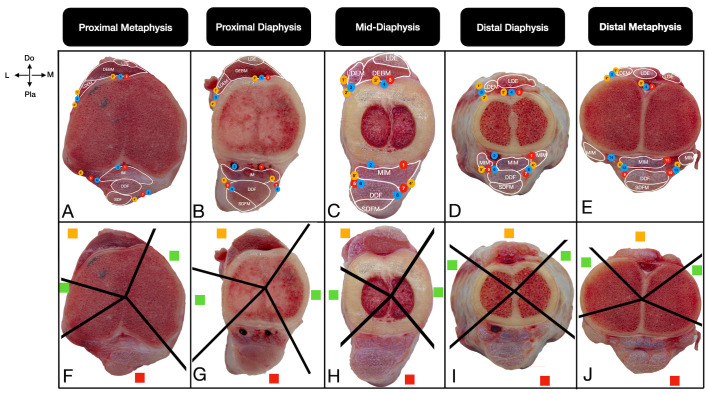
Transverse cross-sectional views of the anatomical (**A**–**E**) and pin placement corridors (**F**–**J**) in the metatarsal bone of the calf at the proximal metaphysis (**A**,**F**), proximal diaphysis (**B**,**G**), mid-diaphysis (**C**,**H**), distal diaphysis (**D**,**I**), and distal metaphysis (**E**,**J**). The straight lines (**F**–**J**) show the boundaries of the pin placement corridors. The red squares indicate unsafe corridors, the yellow squares indicate hazardous corridors, and the green squares indicate safe corridors. Do: Dorsal, Pla: Plantar, M: Medial, L: Lateral. For a complete list of anatomical abbreviations, please refer to Supplementary Table S1.

The corridors were defined on the basis of the presence of musculotendinous units and important neurovascular structures. Safe corridors were defined as longitudinal regions through which pins can be safely inserted, as they contain neither musculotendinous units nor important neurovascular structures. In these zones, the bone is covered primarily by the skin and fascia. Hazardous corridors were defined as regions that have musculotendinous units but no important neurovascular structures. Unsafe corridors were defined as regions that contain both musculotendinous units and important neurovascular structures [19,20,21]. Corridor boundaries were determined qualitatively through direct macroscopic observation of the intermuscular fascial planes and anatomical margins of the muscle bellies relative to the bone cortex. For practical standardization across all sections, the boundary transitioning from a ‘safe’ to a ‘hazardous’ corridor was strictly defined as the exact anatomical point where the first musculotendinous unit encountered the bone surface. Furthermore, the cross-sectional assessments from the right limbs were systematically cross-validated against the topographic dissections of the contralateral left limbs to confirm structural identities. To ensure observational reliability and consistency, these macroscopic evaluations and the final determination of the corridor boundaries were achieved through a consensus between two researchers. Once the corridors had been identified in all cross-sections according to the criteria, their boundaries were physically marked by inserting 21-G cannulas towards the bone cortex. These marked sections were subsequently photographed. For optimal visual clarity in the final figures, solid black lines were digitally superimposed directly over the physical trajectories of the cannulas to clearly demarcate the corridors (e.g., Figure 2, Figure 3, Figure 4 and Figure 5). These 21-G cannulas were utilized strictly as thin, non-destructive visual boundary markers to preserve the delicate fascial planes during qualitative topographical evaluation.

### 2.5. Radius-Ulna

The most proximal and distal transections of the antebrachium were performed at the levels of the proximal and distal radial metaphysis, respectively. The subsequent three sections were distributed equally along the radial diaphysis. Evaluations of the epiphyses and physes of the bones were deliberately excluded from this assessment (Figure 1A,B).

### 2.6. Metacarpus III/IV

The metacarpal bones were transversely sectioned into five levels, starting at the base of the metacarpal tuberosity. The last transection line was placed at least 2 cm proximal to the intercapital incisura to avoid cutting into the metacarpophalangeal joint (Figure 1C,D). The subsequent sections were distributed at equal intervals on the diaphysis of the metacarpal bones.

### 2.7. Tibia

The proximal metaphyseal transection of the tibia was made just below the patellar tendon insertion, and the proximal diaphyseal transection was made at the distal part of the tibial crest. Two more transections were made on the tibial diaphysis. The distal metaphyseal transection was made at least 4 cm proximal to the medial malleolus, taking care that the section did not include the tarsal joint (Figure 1E,F).

### 2.8. Metatarsus

The proximal metaphyseal transection of the metatarsus was located 2 cm distal to the tarso-crural joint, approximately at the level of the base of the metatarsal bone. The proximal, mid, and distal diaphyseal transections were made equally, and the distal metaphyseal section was located 2 cm proximal to the metatarsophalangeal joint (Figure 1G,H).

During the preparation of this manuscript, generative AI technology (Google Gemini; Google LLC, Mountain View, CA, USA) was utilized exclusively for language polishing, grammar correction, and structural formatting to enhance readability. The AI was not involved in data generation, statistical analysis, or the formulation of scientific conclusions.

## 3. Results

### 3.1. Radius-Ulna

#### 3.1.1. Proximal Metaphysis and Proximal Diaphysis

The proximal antebrachium lacks any safe pin placement corridors (Figure 2A,F). The lateral surface is occupied by the lateral digital extensor muscle, while the medial aspect houses the brachialis muscle and critical median artery and vein. A craniolaterally located hazardous corridor and a caudomedially located unsafe corridor make it impossible to pass a pin without penetrating musculotendinous or neurovascular structures. From an anatomical perspective, any theoretical craniolateral direction for half-pins at the proximal metaphysis must pass strictly between muscle groups to avoid musculotendinous damage. Furthermore, a proximomedial direction is anatomically hazardous for full pin penetration because of the high risk of intersecting the median artery (Figure 2B,G).

#### 3.1.2. Mid-Diaphysis

Anatomically, a craniomedial safe corridor emerges between the median artery and the extensor carpi radialis muscle, which is subcutaneously palpable (Figure 2C,H). However, as stated earlier, pin placement at the mid-diaphysis is not clinically recommended because of the high risk of fracture. Hazardous corridors remain craniolaterally (bordered by the extensor carpi radialis and common digital extensor muscles), and unsafe corridors remain caudomedially. The median neurovascular bundle runs distally in a caudodistal direction beneath the pronator teres muscle.

#### 3.1.3. Distal Diaphysis and Distal Metaphysis

The craniomedial safe corridor continues distally. In addition, a second safe corridor appears craniolaterally at the distal diaphysis and metaphysis (Figure 2I,J), between the lateral border of the extensor carpi radialis and the cranial border of the abductor digiti I longus muscle (Figure 2D,E). Hazardous corridors are confined to the lateral and cranial aspects. Theoretically, the anatomical window allows for a lateral-to-medial transfixation direction across the antebrachium if the cranially located extensor carpi radialis muscle is completely bypassed (Table 1).

### 3.2. Metacarpus III/IV

The cross-sectional area of the metacarpal bone is largest at the proximal metaphysis, gradually decreases through the diaphysis, and expands again at the distal metaphysis.

#### 3.2.1. Proximal Metaphysis

A limited safe corridor is located dorsally, bounded by the medial tendon of the common digital extensor muscle and the cranial superficial antebrachial artery (Figure 3A,F). At this level, the medial and lateral regions are classified as hazardous corridors, while the entire palmar region of the metacarpal bone constitutes an unsafe corridor (Figure 3F). Anatomically, a mediolateral direction could theoretically accommodate half or full pins if the direction remains dorsal to the lateropalmar region to prevent intersection with the cranial interosseous artery and the dorsal branch of the ulnar nerve.

#### 3.2.2. Proximal and Mid-Diaphysis

Anatomically safe corridors expand on the medial and lateral aspects, with the lateral corridor notably wider (Figure 3G,H). Anatomically, as the lateral digital extensor and flexor tendons course obliquely towards the dorsolateral aspect, the lateral safe corridor angle progressively decreases from the proximal diaphysis to the mid-diaphysis (Figure 3B,C). The palmar surface remains unsafe owing to the interosseous medius muscle.

#### 3.2.3. Distal Diaphysis and Distal Metaphysis

As the flexor tendons and associated neurovascular branches (common dorsal digital vein III, superficial branch of radial nerve, and superficial antebrachial cranial artery) shift dorsolaterally at the distal diaphysis (Figure 3D), the dorsomedial safe corridor widens, while the lateral safe corridor narrows (Figure 3I). The mediolateral axis theoretically provides an anatomical window for full pin insertion. However, trajectories directed dorsolaterally or palmarly intersect with the flexor tendons, rendering them anatomically unsafe. At the distal metaphysis, the flexor tendons move dorsally, accompanying vascular supplies between the medial and lateral tendons of the common digital extensor muscle (Figure 3E,J) (Table 2).

### 3.3. Tibia

The cross-sectional surface of the proximal tibia is predominantly triangular, transitioning to an elliptical shape at the diaphysis and becoming more rectangular distally.

#### 3.3.1. Proximal Metaphysis and Proximal Diaphysis

Anatomically, a primary safe corridor exists on the medial aspect of the proximal tibia, bounded by the cranial margin of the cranial tibial muscle and the popliteus muscle. Hazardous corridors are located at the lateral and medial aspects (accommodating the tibial cranial and popliteal muscles) (Figure 4A,B). The laterocaudal and caudolateral aspects remain entirely unsafe corridors owing to the presence of major neurovascular structures, particularly the cranial tibial artery and vein (Figure 4F,G). Theoretically, while the medial safe corridor allows for pin placement, the triangular cross-section of the proximal tibia dictates that any mediolateral pin direction must remain adequately caudal to ensure optimal bone purchase without encroaching on the caudolateral unsafe zone.

#### 3.3.2. Mid-Diaphysis

The medial safe corridor persists and widens slightly as the popliteus muscle tapers. Anatomically, a hazardous corridor is located craniolaterally, bounded by the medial edge of the cranial tibial muscle and the cranial edge of the cranial tibial artery and vein (Figure 4C). The caudolateral aspect remains strictly unsafe (Figure 4H).

#### 3.3.3. Distal Diaphysis and Distal Metaphysis

The medial safe corridor continues distally. While the caudolateral aspect of the tibia is generally classified as an unsafe corridor throughout the proximal and mid-sections (Figure 4I,J), a second, narrow safe corridor theoretically emerges at the caudolateral aspect of the distal metaphysis, dividing the caudal unsafe zone into two parts (Figure 4J). The craniolateral region remains an unsafe corridor, as it consists of critical structures, such as the cranial tibial artery and vein, deep and superficial fibular nerves, and associated extensor tendons (Figure 4D,E). Theoretically, the anatomical window allows for medial-to-caudolateral transfixation, provided the direction strictly avoids craniolateral neurovascular bundles (Table 3).

### 3.4. Metatarsus III/IV

The cross-sectional surface area of the metatarsal bone is widest at the proximal metaphysis, narrows significantly at the mid-diaphysis, and expands again towards the distal metaphysis. Throughout all sections, the entire dorsal surface of the metatarsus is a hazardous corridor (encompassing the lateral digital extensor, long digital extensor, and short digital extensor tendon), while the plantar surface is strictly an unsafe corridor (occupied by the interosseous muscle, flexor tendons, and plantar metatarsal vessels).

#### 3.4.1. Proximal Metaphysis

Anatomically safe corridors are located on the medial and lateral aspects of the metatarsal bone, with the medial corridor generally wider. The boundaries of the medial safe corridor are defined by the medial margin of the short digital extensor tendon and interosseous muscle (Figure 5A). The lateral safe corridor is situated between the craniolateral margin of the lateral digital extensor tendon and lateral plantar nerve (Figure 5F). This configuration theoretically accommodates a mediolateral pin direction when it remains strictly central to avoid the dorsally located deep fibular nerve/dorsal metatarsal vessels and the plantarly located flexor tendon complex.

#### 3.4.2. Proximal Diaphysis and Mid-Diaphysis

The medial and lateral anatomically safe corridors expand as the lateral digital extensor tendon courses more dorsally (Figure 5G,H). The dorsal aspect remains hazardous, and the angle of the plantar unsafe corridor increases significantly owing to the division of the interosseous muscle tendon (Figure 5B,C).

#### 3.4.3. Distal Diaphysis and Distal Metaphysis

The lateral and medial safe corridors continue but narrow progressively as the interosseous muscle tendons shift from a plantar position laterally and medially (Figure 5D,E). The dorsal surface remains hazardous, while the plantar unsafe corridor reaches its widest angle at the distal metaphysis (Figure 5I,J). Anatomically, a strict mediolateral transfixation window exists, but any direction deviating dorsally or plantarly poses a severe risk of penetrating the extensor or flexor musculotendinous complexes and their associated neurovascular networks (Table 4).

## 4. Discussion

This study establishes a comprehensive anatomical map of safe, hazardous, and unsafe pin placement corridors for the distal long bones of the forelimbs and hindlimbs of neonatal Simmental calves. To the best of our knowledge, no previous ex vivo cross-sectional anatomical mapping of the long bones has been performed specifically for neonatal calves, an age group in which ESF is frequently applied [5]. Therefore, the novel contribution of this study is filling this critical gap in the veterinary literature by providing the first systematic, level-by-level topographic reference. The Simmental breed was selected for this study owing to its high prevalence in our region. Limb fractures in these animals are prevalent, often arising from dystocia interventions or accidental trauma. While ESF is a cost-effective and versatile method in bovine orthopaedics, successful clinical outcomes rely on precise topographic knowledge to avoid iatrogenic damage to vital neurovascular and musculotendinous structures [1,8,9,22].

Radioulnar fractures account for approximately 6% of fractures in neonatal calves [1]. Various ESF techniques, including type II, circular ESF, and transfixation pinning and casting, have been used to manage these fractures [11,13,22,23]. In these clinical reports, full transfixation pins were placed at various levels and in different trajectories, such as lateral to medial, craniolateral to caudomedial, and craniomedial to caudolateral [11,13,23]. Although our anatomical mapping demonstrates the absence of a completely safe corridor at the first two proximal levels of the antebrachium, no major neurovascular complications have been explicitly reported in the bovine literature after ESF application in this region.

However, in small animal orthopaedics, life-threatening haemorrhage at the medio-proximal pin site due to delayed median artery rupture following radioulnar ESF application has been documented in dogs [24,25]. Although such a specific vascular complication has not been formally documented in calves, our cross-sectional mapping demonstrates that the median artery occupies a similarly vulnerable position at the medio-proximal aspect of the bovine radius. Therefore, from a strictly anatomical perspective, if pin placement in the proximal radius is required, a lateral-to-craniomedial direction through the brachialis muscle provides a structural pathway to help bypass this major arterial location.

However, a critical clinical distinction must be made regarding the radius. While mid-diaphyseal levels in the radius appear anatomically safe owing to the absence of major neurovascular structures, pin placement at these levels is clinically discouraged because this increases stress in these locations, significantly increasing the risk of iatrogenic fractures in weight-bearing neonatal calves [6]. Therefore, although these zones are described anatomically, in the radius it is clinically advisable to prioritize the metaphyseal regions to maintain biomechanical integrity.

Tibial fractures constitute approximately 8.8% of limb fractures in neonatal calves [1]. These fractures are managed using circular, linear, and epoxy-pin ESF, which have been well documented in the literature [11,13,26,27]. Our cross-sectional analysis revealed that the tibia provides a continuous safe corridor for half-pin application along its entire medial surface at all evaluated levels. The boundaries of this extensive safe zone are defined by important musculotendinous and neurovascular structures located cranially, caudally, and laterally. When full transfixation pins are required, mediolateral pin placement is anatomically viable. However, strict attention is necessary to protect the tibial artery, which is located at the caudolateral aspect of the bone. Furthermore, structural and geometric considerations are paramount in proximal regions. As the transverse cross-section of the tibia is distinctly triangular at the first two proximal levels, any mediolateral pin placement must be positioned adequately close to the caudal edge to ensure optimal cortical bone purchase.

In the metacarpal and metatarsal bones, the anatomical configuration generally allows for versatile pin placements, with safe corridors expanding on both the medial and lateral aspects from the diaphysis to the distal metaphysis [7,9]. In contrast to the metatarsus, which safely accommodates mediolateral pin placement, even at its proximal metaphysis, the corresponding region in the metacarpus presents a more constrained environment. At this specific metacarpal level, although a completely safe corridor is restricted to a narrow dorsal window, the medial and lateral regions are classified as hazardous. Consequently, despite the limited dorsal safe zone, the presence of these bilateral hazardous corridors theoretically permits the application of full transfixation pins in a mediolateral direction. This is clinically viable if the pin direction remains dorsal to the strictly unsafe palmar region, thereby avoiding intersection with the cranial interosseous artery and dorsal ulnar nerve.

In clinical practice, achieving pin placement solely through safe corridors is not always feasible. Hazardous corridors, defined by the presence of musculotendinous units but the absence of major neurovascular structures, are viable alternatives [19,20,21]. Although pin insertion through these areas carries the risk of penetrating muscle bellies, previous clinical studies indicate that the damage caused by pins with relatively small diameters is generally well tolerated and does not typically result in significant functional loss [11,13,28,29,30]. While previous clinical reports have demonstrated the successful use of ESF in bovine orthopaedics [2,13,30,31,32], a direct, level-by-level comparison between our anatomically defined corridors and historical pin placement sites was not feasible. A review of the existing veterinary literature reveals a significant lack of standardized reporting regarding the specific anatomical levels and exact trajectories of transcortical pins. Most clinical studies provide only representative clinical photographs or postoperative radiographs from a limited number of cases without explicitly detailing the precise anatomical insertion points. Consequently, this study not only maps these vital corridors but also highlights the need for more rigorous and standardized anatomical reporting of ESF applications in future clinical trials.

The anatomical configuration of safe, hazardous, and unsafe corridors strongly influences the selection of the ESF frame type (e.g., Type I versus Type II or circular fixators) and the use of half or full transfixation pins. A significant limitation occurs when an anatomically safe corridor is diametrically opposed to an unsafe corridor; in such configurations, placing a full pin through the bone is strictly contraindicated. For instance, in the proximal metaphysis of the radius, the presence of major neurovascular structures on the opposite cortex restricts the surgeon to using half-pins [24,25], thereby limiting the construct to a unilateral Type I ESF frame [10,11,13]. Conversely, when a safe corridor is opposite another safe or hazardous zone, full pins can be safely applied, allowing for biomechanically stiffer bilateral frames, such as Type II or circular ESF systems. In clinical practice, transfixation for the metacarpus and metatarsus is routinely performed in a mediolateral direction rather than craniocaudally/dorsoplantarly. Since our mapping indicates that the medial and lateral aspects of these bones generally present safe or hazardous corridors, bilateral pin placement is highly viable in these regions. Similarly, because the tibia provides a continuous safe corridor on its medial aspect, mediolateral full pins can be successfully applied to construct these stiffer systems if the trajectory is directed slightly cranially towards the lateral hazardous corridor, meticulously avoiding the caudolateral unsafe zones. Ultimately, these anatomical restrictions emphasize that a surgeon’s deep knowledge of cross-sectional anatomy is the primary safeguard against iatrogenic injury, enabling precise pin placement and guiding the appropriate selection of the ESF construct for neonatal calves.

A primary limitation of this study is its ex vivo anatomical design and the relatively small sample size of six calves. To establish a standardized and reproducible topographic baseline, our sample was restricted to male Simmental calves of similar age (1–2 months) and body weight, thereby minimizing inter-individual variability. This specific demographic was deliberately targeted because long bone fractures are most frequent in this early postnatal period due to dystocia. Specific cross-sectional levels were selected to map the musculotendinous and neurovascular structures and establish an anatomical baseline rather than to provide definitive clinical pin application sites. Accordingly, the assessment of safe, hazardous, and unsafe corridors was based on direct macroscopic topographic observations rather than quantitative angular or morphometric measurements, thereby precluding complex statistical analysis. Therefore, the primary clinical implication of this study is to serve as a helpful anatomical guide for pin placement in clinical practice, rather than to present absolute surgical rules. Veterinary surgeons should integrate this anatomical guidance with specific fracture configurations, mechanical requirements, and the individual patient’s conformation. Furthermore, our methodology involved freezing the limbs in a hanging position, which resulted in a full extension that potentially exceeded physiological limits and caused slight shifts in muscle locations compared with a natural position. Finally, extrapolating these anatomical corridors to older calves requires caution. It is highly probable that safe corridor boundaries do not scale purely proportionally with somatic growth; differential development rates of bone versus muscle mass, alongside shifting relative positions of neurovascular bundles with age, could significantly alter these topographic windows. While neonatal Simmental calves were selected because of their regional prevalence, potential differences arising from interindividual variability, sex, body weight, and breed-specific muscular development must be carefully considered when generalizing these results.

In conclusion, this study provides a comprehensive anatomical map of safe, hazardous, and unsafe pin placement corridors for ESF in the forelimbs and hindlimbs of neonatal Simmental calves. It must be emphasized that these data are purely anatomical; consequently, inferences regarding the clinical viability and tissue tolerability of pin placement through specific corridors are indirect and based on existing literature rather than empirical in vivo testing. Nevertheless, a profound understanding of cross-sectional topography is essential to prevent iatrogenic damage to vital neurovascular and musculotendinous structures during fracture management. Provided that biomechanical integrity is maintained (e.g., prioritizing metaphyseal over diaphyseal regions), anatomically safe corridors represent the most optimal pathways for pin insertion. When necessary in fracture configuration, hazardous corridors can be utilized as viable alternatives if pins are meticulously placed between muscle groups to minimize soft tissue trauma. Conversely, our anatomical mapping indicates that unsafe corridors carry severe risks of iatrogenic trauma to major neurovascular bundles and critical tendinous structures, and thus, transcortical pinning through these zones is anatomically contraindicated. Ultimately, while this topographic mapping provides a fundamental anatomical guide, it should not be applied in a purely algorithmic way. Veterinary surgeons must meticulously integrate these anatomical recommendations with specific fracture configurations, mechanical requirements, and the individual patient’s conformation to determine the final pin placement sites and ESF construct.

## Figures and Tables

**Table 1:** Direction of safe, hazardous and unsafe corridors in transversal sections of the calf radius-ulna.

Levels of Radius and Ulna	Safe Corridor Direction	Hazardous Corridor Direction	Unsafe Corridor Direction
Proximal metaphysis	N/A	Craniolateral	Caudomedial
Proximal diaphysis	N/A	Craniolateral	Caudomedial
Mid-diaphysis	Medial	Craniolateral	Caudal
Distal diaphysis	Medial and Craniolateral	Cranial and Lateral	Caudal
Distal metaphysis	Medial and Craniolateral	Cranial and Lateral	Caudal

N/A: unavailable.

**Table 2:** Direction of safe, hazardous and unsafe corridors in transversal sections of the calf metacarpal bones.

Levels of Metacarpal Bones	Safe Corridor Direction	Hazardous Corridor Direction	Unsafe Corridor Direction
Proximal metaphysis	Dorsolateral	Dorsomedial and Lateral	Palmar
Proximal diaphysis	Lateral and Medial	Dorsolateral	Palmar
Mid-diaphysis	Lateral and Medial	Dorsolateral	Palmar
Distal diaphysis	Lateral and Dorsomedial	Dorsolateral	Palmar
Distal metaphysis	Lateral and Medial	Dorsal	Palmar

**Table 3:** Direction of safe, hazardous and unsafe corridors in transversal sections of the calf tibia.

Levels of Tibia	Safe Corridor Direction	Hazardous Corridor Direction	Unsafe Corridor Direction
Proximal metaphysis	Craniomedial	Caudomedial and Lateral	Caudal
Proximal diaphysis	Medial	Caudomedial and Craniolateral	Caudolateral
Mid-diaphysis	Medial	Craniolateral	Caudolateral
Distal diaphysis	Medial	Cranial	Caudolateral
Distal metaphysis	Medial and Caudolateral	Cranial	Caudal and Craniolateral

**Table 4:** Direction of safe, hazardous and unsafe corridors in transversal sections of the calf metatarsal bones.

Levels of Metatarsal Bones	Safe Corridor Direction	Hazardous Corridor Direction	Unsafe Corridor Direction
Proximal metaphysis	Lateral and Medial	Dorsolateral	Plantar
Proximal diaphysis	Lateral and Medial	Dorsolateral	Plantar
Mid-diaphysis	Lateral and Medial	Dorsal	Plantar
Distal diaphysis	Lateral and Medial	Dorsal	Plantar
Distal metaphysis	Lateral and Medial	Dorsal	Plantar

## Data Availability

The original contributions presented in this study are included in the article/Supplementary Material. Further inquiries can be directed to the corresponding author.

## Supplementary Materials

The following supporting information can be downloaded at: https://www.mdpi.com/article/10.3390/vetsci13050475/s1. Table S1: Full list of anatomical abbreviations and neurovascular numerical codes used in Figure 2, Figure 3, Figure 4 and Figure 5.
