# Supplementary material for: Determination of Safe Corridors for External Fixation Pin Insertion in the Distal Long Bones: An Ex Vivo Anatomical Study in Neonatal Simmental Calves
Source: Vet Sci. 2026 May 14;13(5):475. doi: 10.3390/vetsci13050475 (PMC13211464; doi:10.3390/vetsci13050475)
Supplement: Supplementary file 1 [file vetsci-13-00475-s001.zip › Supplementary Table S1.pdf]

Supplementary Table S1. Full list of anatomical abbreviations and neurovascular numerical codes used in Figures 2–5.

Figure and Anatomical Region	Abbreviation and Codes	Full Anatomical Structure
**FIGURE 2: Radius-Ulna**	**ADL**	Abductor digiti I longus muscle
	**BM**	Brachialis muscle
	**ECR**	Extensor carpi radialis muscle
	**EDC**	Common digital extensor muscle
	**EDC'**	Medial head of common digital extensor muscle (Extensor digiti III)
	**EDC***	Lateral head of common digital extensor muscle
	**EDL**	Lateral digital extensor muscle
	**FCR**	Flexor carpi radialis muscle
	**FCU**	Flexor carpi ulnaris muscle
	**FDP**	Deep digital flexor muscle
	**FDP'**	Deep digital flexor muscle (ulnar head)
	**FDP***	Deep digital flexor muscle (radial head)
	**FDS**	Superficial digital flexor muscle
	**PT**	Pronator teres muscle
	**TPM**	Transverse pectoral muscle
	**1'**	Medial cutaneous antebrachial nerve
	**2'**	Cranial cutaneous antebrachial nerve
	**3'**	Superficial branch of radial nerve
	**4'**	Ulnar nerve
	**5'**	Median nerve
	**1**	Cephalic vein
	**2**	Median vein
	**3**	Median artery
**FIGURE 3: Metacarpus**	**ECR**	Extensor carpi radialis muscle
	**EDCL**	Lateral tendon of common digital extensor muscle
	**EDCM**	Medial tendon of common digital extensor muscle
	**FCR**	Flexor carpi radialis muscle
	**FCU**	Flexor carpi ulnaris muscle
	**FDP**	Deep digital flexor muscle
	**FDS**	Superficial digital flexor muscle
	**IM**	Interosseous muscle (Interosseus medius)
	**LDE**	Lateral digital extensor tendon
	**1'**	Superficial branch of radial nerve
	**2'**	Palmar branch of ulnar nerve
	**3'**	Dorsal branch of ulnar nerve
	**4'**	Median nerve
	**1**	Common dorsal digital vein III
	**2**	Cranial superficial antebrachial artery
	**3**	Cranial interosseous artery
	**4**	Radial vein
	**5**	Radial artery
	**6**	Median artery
	**7**	Median vein
**FIGURE 4: Tibia**	**BFM**	Biceps femoris muscle

	**DDFM**	Deep digital flexor muscle
	**FLM**	Fibularis (peroneus) longus muscle
	**FTM**	Fibularis (peroneus) tertius muscle
	**LDFM**	Long digital flexor muscle (medial head of deep digital flexor)
	**LED**	Long digital extensor muscle
	**LED'**	Medial belly of long digital extensor muscle (Extensor digiti III)
	**LEDM**	Lateral digital extensor muscle
	**LGM**	Lateral head of gastrocnemius muscle
	**MGM**	Medial head of gastrocnemius muscle
	**PM**	Popliteus muscle
	**SDFM**	Superficial digital flexor muscle
	**STM**	Semitendinosus muscle
	**TCM**	Cranial tibial muscle
	**1'**	Common fibular (peroneal) nerve
	**2'**	Saphenous nerve
	**3'**	Tibial nerve
	**1**	Cranial tibial artery
	**2**	Cranial tibial vein
	**3**	Saphenous artery
	**4**	Medial saphenous vein
**FIGURE 5: Metatarsus**	**DDF**	Deep digital flexor tendon
	**DEBM**	Short digital extensor muscle (Extensor digitorum brevis)
	**IM**	Interosseous muscle (Interosseus medius)
	**LDE**	Long digital extensor muscle
	**LDEM**	Lateral digital extensor muscle
	**SDF**	Superficial digital flexor tendon
	**SDFM**	Superficial digital flexor muscle
	**1'**	Lateral branch of superficial fibular nerve
	**2'**	Medial branch of superficial fibular nerve
	**3'**	Deep fibular nerve
	**4'**	Common plantar digital nerve IV
	**5'**	Lateral plantar nerve
	**1**	Plantar metatarsal artery
	**2**	Plantar metatarsal vein
	**3 blue**	Lateral saphenous vein
	**3 red**	Dorsal metatarsal artery
	**4**	Dorsal metatarsal vein
	**5**	Lateral plantar artery
	**6**	Lateral plantar vein
	**7**	Medial plantar artery
	**8**	Medial plantar vein
	**9**	Common plantar digital artery IV
	**10**	Common plantar digital artery III
	**11**	Common plantar digital artery II
	**12**	Common plantar digital vein IV
	**13**	Common plantar digital vein III

	**14**	Common plantar digital vein II
